# Sustainable Crop Protection, Global Climate Change, Food Security and Safety—Plant Immunity at the Crossroads

**DOI:** 10.3390/vaccines8010042

**Published:** 2020-01-24

**Authors:** Marcello Iriti, Sara Vitalini

**Affiliations:** Department of Agricultural and Environmental Sciences, Milan State University, 20133 Milan, Italy; sara.vitalini@unimi.it

**Keywords:** plant protection products, agrochemicals, systemic acquired resistance, invasive species, coevolution, pesticide residues, mycotoxins, fungicide resistance

## Abstract

The development of novel strategies of plant disease management is crucial in view of the growing demand of sustainability in agri-food chains. The use of agrochemicals is not without risk for the consumer and environment in terms of their residues in food, feed, water bodies and harmful effects on nontarget organisms. However, because of the high global annual yield losses attributable to plant diseases and also due to global climate changes that have exacerbated some phytosanitary emergences, chemical input in agriculture is mandatory. In this complex scenario, the use of agrochemicals that boost the plant immune system represents a relatively novel approach in crop protection. These plant protection products are not antimicrobial or fungicidal agents, but include both natural and synthetic elicitors and plant activators that only target the host immune system, with no biocide mechanism of action. In general, these products present a number of strengths: they leave no residue and should not select resistant pathogen strains, they can be used to control virus diseases, and can increase the levels of bioactive phytochemicals in plant foods.

At a first reading of the title of this brief commentary, one could think about the correlation between sustainable crop protection, global climate change, food security and safety, and plant immunity.

*First*, a major health concern associated with agricultural intensification is the increased use of pesticides. Directive 2009/128/EC of the European Council establishes the sustainable use of pesticides by reducing the risks and impacts of pesticide use on human health and the environment and promoting the use of integrated pest management and alternative approaches or techniques such as non-chemical alternatives to pesticides. These measures are complementary to Regulation (EC) No. 396/2005, which declares a high level of consumer protection needs to be ensured, with provisions relating to maximum levels of pesticide residues in food and feed of plant and animal origin.

*Second*, global climate change is the result of anthropogenic emissions of greenhouse gases in recent decades, the highest in history. As a result, the atmosphere and ocean have warmed, the amounts of snow and ice have decreased, and the sea level has risen. Human activities are estimated to have caused approximately 1.0 °C of global warming above pre-industrial levels that is likely to reach 1.5 °C by 2050 if it continues to rise at the current rate. These climate changes have caused and will cause impacts on human and natural systems, if not directly then via an increased rate of extreme weather and climate events [1]. Global warming and climate change have altered the distribution areas of many plant, animal and microbial species, with the entry of typically North African species in the Mediterranean area or of Mediterranean species in the continental European area, thus altering the coevolution between native host plants and alien parasites. Coevolution is a process of reciprocal selective pressure and adaptation among ecologically interacting species; it is not only relevant in host–parasite systems. According to the Global Invasive Species Database of the International Union for Conservation of Nature (IUCN), three plant pathogens are listed among ‘100 of the World’s Worst Invasive Alien Species’. *Cryphonectria parasitica*, the fungal causal agent of chestnut blight; *Ophiostoma novo-ulmi*, the fungal causal agent of Dutch elm disease; and *Phytophthora cinnamomi*, the oomycete causing dieback, crown and root rot in many hundreds of woody perennial species. The list also includes the whitefly *Bemisia tabaci*, the vector that transmits over a hundred viruses to many hundreds of plant species including Cotton Leaf Curl Virus, Tomato Yellow Leaf Curl Virus and Cucumber Vein Yellowing Virus [2] (http://www.iucngisd.org/gisd/100_worst.php). These data are in agreement with the European Alien Species Information Network [3]. Relevant and recent cases of devastating alien plant parasites include the pine wood nematode *Bursaphelenchus xylophilus* [4] and the bacterium *Xylella fastidiosa* [5], both endemic of the Americas that still represent phytosanitary emergencies. The former is a major threat to European forests, with critical outbreaks in Portugal and Spain, whereas *X. fastidiosa* is the causal agent of Olive Quick Decline Syndrome, the devastating disease destroying olive trees in southern Italy. Of note, both parasites are vectored by arthropods.

*Third*, since 1950, the world population has increased from about 2.5 billion to more than 7 billion people, and is expected to exceed 9 billion by 2050. In this perspective, healthy and safe food of high nutritional quality will have to be adequately secured for the growing population, in an environmentally sustainable manner. To meet this growing demand, food production is expected to have to rise by a further 70–100% by 2050. Therefore, even if food security can simply be defined as the adequate access to food in both quality and quantity, four main dimensions of food security have been identified in the last two decades. These include: (1) food availability (the ‘supply side’)—the physical availability of sufficient quantities of food of appropriate quality, determined by the levels of domestic food production, stocks, imports and trade; (2) food access (the ‘economic side’)—mostly depending on incomes and prices; (3) food utilization—utilization of food through adequate diet, clean water, sanitation and healthcare to reach a state of nutritional well-being where all physiological needs are met; and (4) stability of the other three dimensions over time, i.e., access to adequate food at all times (not on a periodic basis) because adverse climatic conditions, protracted political crises and economic instability may have an impact on food security, deteriorating the people nutritional status [6]. Among these pillars, the first one (food availability) is the most dependent on plant health. Indeed, the estimated potential annual yield losses caused by plant pathogens are up to 16% globally [7]. (Oerke, 2006).

*Fourth*, climate changes have also influenced the incidence of toxigenic fungi in Europe due to an alteration of the host–pathogen interaction as well as optimal conditions of temperature and humidity, predisposing fungal colonization and mycotoxin production [8]. Mycotoxins enter the food chain as a result of pre- and/or post-harvest fungal infections of crops and are typically found in cereals, dried fruits, nuts, spices and some beverages such as wine, coffee and beer. Mycotoxin contamination of food and feed represents a global threat for human and animal health because of their hepatotoxicity, nephrotoxicity, genotoxicity and carcinogenicity in addition to being immunosuppressant agents and endocrine disruptors. The most common mycotoxins that pose a concern to human and animal health include aflatoxins, ochratoxins and fusarial toxins (trichothecenes, fumonisins and zearalenone) mainly produced by *Aspergillus* spp., *Penicillium* spp. and *Fusarium* spp. with the role of secondary metabolites. In particular, the risk of aflatoxin contamination in corn markedly increased in south and central Europe in the last decade due to favorable climatic conditions for the growth of *A. flavus* (the main *Aspergillus* species producing aflatoxins). Similarly, the profile of mycotoxigenic *Fusarium* species associated with wheat is in continuous change in Europe, with an alarming rising contamination of *F. graminearum* in central and northern Europe [8]. According to the Rapid Alert System for Food and Feed [9], 655 notifications concerned mycotoxin contamination of food and feed in 2018, ranking second in the top 10 hazard categories in the EU. Noteworthy, pesticide residues in food and feed ranked third (Table 1).

*The crossroad.* Plant innate immunity consists of two different recognition systems to perceive parasites, pattern-triggered immunity (PTI, formerly known as non-host resistance) and effector-triggered immunity (ETI, previously named host resistance). Highly conserved pathogen-associated molecular patterns (PAMPs), microbe-associated molecular patterns (MAMPs) and herbivore-associated molecular patterns (HAMPs) are perceived by membrane pattern recognition receptors (PRRs), thus activating PTI, as well as endogenous damage-associated molecular patterns (DAMPs) released by the damaged host cells and tissues. In other words, plants are able to recognize and distinguish among self, non-self and altered self. Therefore, an entire taxonomic group of pathogens featuring a particular PAMP (e.g., bacterial flagellin or fungal chitin) can be recognized by a specific PRR. Receptor-like kinases and receptor-like proteins are the typical PRRs in plants. Effector proteins encoded by avirulence (*avr*) genes and secreted by pathogens into host cells trigger ETI, which are in turn recognized by intracellular nucleotide-binding domain leucine-rich repeat (NLR)-type receptors encoded by resistance (*R*) genes. This phenomenon was formerly described in the gene-for-gene model typical of race-specific resistance of incompatible interactions. Downstream to recognition, common plant defense reactions include oxidative and nitrosative burst (i.e., reactive oxygen and nitrogen species production) and the hypersensitive response (a form of programmed cell death) at the attempted penetration site. In addition to these local and transient immune responses at the infection site, plants can activate systemic acquired resistance (SAR), a long lasting, broad-spectrum and nonspecific immunity in uninfected tissues that also potentiates the host resistance to subsequent pathogen attacks. Biosynthesis of phytoalexins (antimicrobial secondary metabolites) and accumulation of pathogenesis-related proteins in distal tissues are typical systemic defense responses associated with a local and systemic increase of salicylic acid levels [10,11].

Plant innate immunity (particularly SAR) can be induced with elicitors and plant activators that represent relatively novel targets for the development of commercial agrochemicals or plant protection products. Elicitors can be divided into biotic and chemical elicitors, whereas the term ‘plant activator’ is more general, including both synthetic and natural elicitors. Biotic elicitors derive from living organisms such as laminarin from brown algae, chitosan from fungi and crustaceans or mild/weak phytovirus strains, while chemical elicitors include functional analogues of salicylic acid such as benzothiadiazole [12]. A number of active substances are registered as elicitors and plant activators in the European Union (Table 2). The use of these products in crop protection is revolutionary: they are not antimicrobial agents as they are based on a non-biocide mechanism of action that only target the plant host immune system.

Undoubtedly, plant disease management with SAR inducers presents a number of strengths (Table 2 and Table 3). In general, compared with fungicides, elicitors and plant activators are nontoxic, environmentally friendly and not classified according to the Globally Harmonized System of Classification and Labelling of Chemicals. In addition, minimum residue levels in food are not required and toxicological information, i.e., acceptable daily intake (ADI), acute reference dose (ARfD) and acute operator exposure level (AOEL) are not applicable. Priming the plant immune system can also represent a strategy to control viral and bacterial diseases that are incurable in plants, as well as to confer tolerance to some abiotic stresses such as water deficit [15]. Indeed, some elicitors such as chitosan stimulate hormone-dependent abscisic acid (ABA)-induced stomatal closure, a recognized immune mechanism at the preinfectional level that also limits water loss in drought conditions, a process relevant in a global climate change scenario [16,17]. Chitosan is a deacetylated derivative of chitin, the structural component of the fungal cell wall and the insect exoskeleton, which is recognized as a PAMP by the plant perception system. It was also shown to reduce the severity of Fusarium Head Blight Disease in cereals and associated deoxynivalenol (a trichothecene mycotoxin) contamination of grain [18]. In addition, the biosynthesis of jasmonic acid and other oxylipins is increased by chitosan via the octadecanoid pathway. Jasmonic acid is a signal molecule activating plant resistance against insects and necrothrophic fungi in crosstalk with the ethylene signaling pathway [19,20].

Intriguingly, treatment with SAR inducers that stimulate plant secondary metabolism (in particular the biosynthesis of phytoalexins) may increase the healthy potential of some plant foods as a kind of biofortification. Indeed, plant defense metabolites include bioactive phytochemicals such as polyphenols, which are recognized as health-promoting components of plant foods [33,34,35]. Finally, and not least, the use of elicitors and plant activators poses no risk of selecting agrochemical resistant pathogen strains because of their mechanism of action (targeting the multigenic defense system of the host plant). Of note, drug (including fungicides, insecticides and herbicides) resistance represents one of the major threats to global health and food security.

## Figures and Tables

**Table 1:** Notifications by type of hazard and product category in 2018 *.

	Type of Notification	Number of Notifications
Type of hazard	Pathogenic microorganisms	979
	Mycotoxins	655
	Pesticide residues	276
	Composition	224
	Allergens	207
	Poor and insufficient controls	179
	Foreign bodies	168
	Food additives and flavourings	142
Product category	Nuts, nut products and seeds	667
	Fruits and vegetables	475
	Fish and fish products	330
	Feed	313
	Poultry and poultry products	265
	Dietetic foods, food supplements and fortified foods	255

* Source: Rapid Alert System for Food and Feed [9].

**Table 2:** Elicitors and plant activators approved in European Union *.

Active Substance	Classification GHS ^‡^	MRLs **	Toxicological Information		
			ADI ^#^ (mg/kg bw/d) ^§^	ARfD ^#^ (mg/kg bw)	AOE ^#^ (mg/kg bw/d)
**Elicitors**					
Chitosan hydrochloride	No classification	No MRL required	NA ^†^	NA	NA
Fructose	No classification	No MRL required	NA	NA	NA
Heptamaloxylglucan	No classification	No MRL required	NA	NA	NA
Laminarin	No classification	No MRL required	NA	NA	NA
Mild Pepino Mosaic Virus isolate VC 1	No classification	No MRL required	NA	NA	NA
Mild Pepino Mosaic Virus isolate VX 1	No classification	No MRL required	NA	NA	NA
Pepino Mosaic Virus strain CH2 isolate 1906	No classification	No MRL required	NA	NA	NA
Sucrose	No classification	No MRL required	NA	NA	NA
Zucchini Yellow Mosaic Virus weak strain	No classification	No MRL required	NA	NA	NA
**Plant activators**					
Acibenzolar-S-methyl (benzothiadiazole)	Skin corrosion/irritation Category 2 (H315) Skin sensitisation Category 1 (H317) Serious eye damage/irritation Category 2 (H319) Specific target organ toxicity single exposure Category 3 (H335) Hazardous to aquatic environment short term/acute Category 1 (H400) Hazardous to aquatic environment long term/chronic Category 1 (H410)	MRLs required ^¥^	0.03	0.03	0.03
Cerevisane	No classification	No MRL required	NA	NA	NA

* Source: EU Pesticide database [13] retrieved on 4 January 2020; adapted from Iriti and Varoni [14]. ^‡^ Globally Harmonized System of Classification and Labelling of Chemicals. ** Minimum Residue Levels. ^#^ ADI, acceptable daily intake; ARfD, acute reference dose; AOEL, acceptable operator exposure level. ^§^ bw, body weight; d, day. ^†^ NA, not applicable. ^¥^ Sum of acibenzolar-S-methyl and acibenzolar acid (free and conjugated).

**Table 3:** Main biological activities of the most investigated plant protection products activating innate immunity and systemic acquired resistance in food plants.

Active Substance	Biological Activities	References
	**Resistance Against Viruses**	
Chitosan	Alfalfa Mosaic Virus/Bean (*Phaseolus vulgaris*)	[21]
Chitosan	Potato Spindle Tuber Viroid/Tomato (*Solanum lycopersicum*)	[22]
Chitosan	Potato Virus X/Potato (*Solanum tuberosum*)	[23]
Chitosan	Tobacco Mosaic Virus/Tobacco (*Nicotiana tabacum*)	[24]
Chitosan	Tobacco Necrosis Virus/Bean	[16]
	**Mycotoxin Contamination**	
Chitosan	Decrease of deoxynivalenol contamination of cereals	[18]
Chitosan	Decreased trichothecene accumulation in potato tubers	[25]
	**Abiotic Stress Tolerance**	
Chitosan	Anti-transpirant activity	[17]
Chitosan	Reduction of stomatal conductance	[26]
	**Secondary Metabolite Biosynthesis**	
Benzothiadiazole	Resveratrol, anthocyanins/Grape (*Vitis vinifera*)	[27]
Benzothiadiazole	Proanthocyanidins/Grape	[28]
Benzothiadiazole	Melatonin/Grape	[29]
Benzothiadiazole	Lycopene/Tomato	[30]
Chitosan	Polyphenols/Grape	[31]
Chitosan	Melatonin/Grape	[32]
